# Incidence and relative risk of peripheral neuropathy in cancer patients treated with eribulin: a meta-analysis

**DOI:** 10.18632/oncotarget.21057

**Published:** 2017-09-19

**Authors:** Ling Peng, Yun Hong, Xianghua Ye, Peng Shi, Junyan Zhang, Yina Wang, Qiong Zhao

**Affiliations:** ^1^ Department of Thoracic Oncology, The First Affiliated Hospital, School of Medicine, Zhejiang University, Hangzhou, Zhejiang Province, China; ^2^ Department of Pharmacy, The First Affiliated Hospital, School of Medicine, Zhejiang University, Hangzhou, Zhejiang Province, China; ^3^ Department of Radiotherapy, The First Affiliated Hospital, School of Medicine, Zhejiang University, Hangzhou, Zhejiang Province, China; ^4^ Department of Medical Statistics, Children’s Hospital of Fudan University, Shanghai, China; ^5^ Center for Evidence-Based Medicine, Fudan University, Shanghai, China; ^6^ Bothwin Clinical Study Consultant, Seattle, WA, USA

**Keywords:** eribulin, peripheral neuropathy, incidence, relative risk, meta-analysis

## Abstract

**Background:**

Eribulin is a microtubule inhibitor, which is approved for the treatment of breast cancer. Peripheral neuropathy has been reported in the studies of eribulin, but the incidence and relative risk (RR) of eribulin-associated peripheral neuropathy varied greatly in cancer patients. The purpose of this meta-analysis was to determine the overall incidence and RR of eribulin-associated peripheral neuropathy in cancer patients.

**Materials and Methods:**

Pubmed database and Embase and abstracts presented at the American Society of Clinical Oncology (ASCO) meetings were systematically reviewed for primary studies. Eligible studies included prospective clinical trials and expanded access programs of cancer patients treated with eribulin. Statistical analyses were performed to calculate the incidences, RRs, and 95% confidence intervals (CIs).

**Results:**

Altogether, 4,849 patients from 19 clinical trials were selected for this meta-analysis. The incidences of all-grade and high-grade peripheral neuropathy were 27.5% (95% CI: 23.3–32.4%) and 4.7% (95% CI: 3.6–6.2%), respectively. The relative risks of peripheral neuropathy of eribulin compared to control were increased for all-grade (RR = 1.89, 95% CI: 1.10–3.25) but not statistically significant for high-grade (RR = 2.98, 95% CI: 0.71–12.42).

**Conclusions:**

The use of eribulin is associated with an increased incidence of peripheral neuropathy. The RR is increased for all-grade peripheral neuropathy.

## INTRODUCTION

Eribulin mesylate (E7389) is a synthetic analog of halichondrin B, which is a nontaxane microtubule inhibitor, isolated from the rare marine sponge *Halichondria okadai* [1]. Eribulin was approved for the treatment of metastatic breast cancer patients who have received two chemotherapeutic regimens previously.

Although eribulin is well-tolerated, significant toxicities are reported with its use. Chemotherapy-induced peripheral neuropathy (CIPN) is one of the major toxicities, which will lead to a significant decrease in the patient’s quality of life. Symptoms of CIPN are symmetric painful paresthesia, numbness, peripheral ataxia, and weakness [2]. Monitoring and management of peripheral neuropathy is of great importance because serious peripheral neuropathy may lead to poor quality of life and dose reduction.

Due to the limited sample size in each primary study, great variation exits concerning the reported incidences of peripheral neuropathy among studies. To determine the incidence and RR of peripheral neuropathy associated with eribulin, we aim to investigate the incidence and the RR of eribulin-associated peripheral neuropathy by performing a meta-analysis. We have also studied potential factors affecting the effect size, including tumor type and trial design.

## RESULTS

### Study selection and characteristics

**Figure 1:**
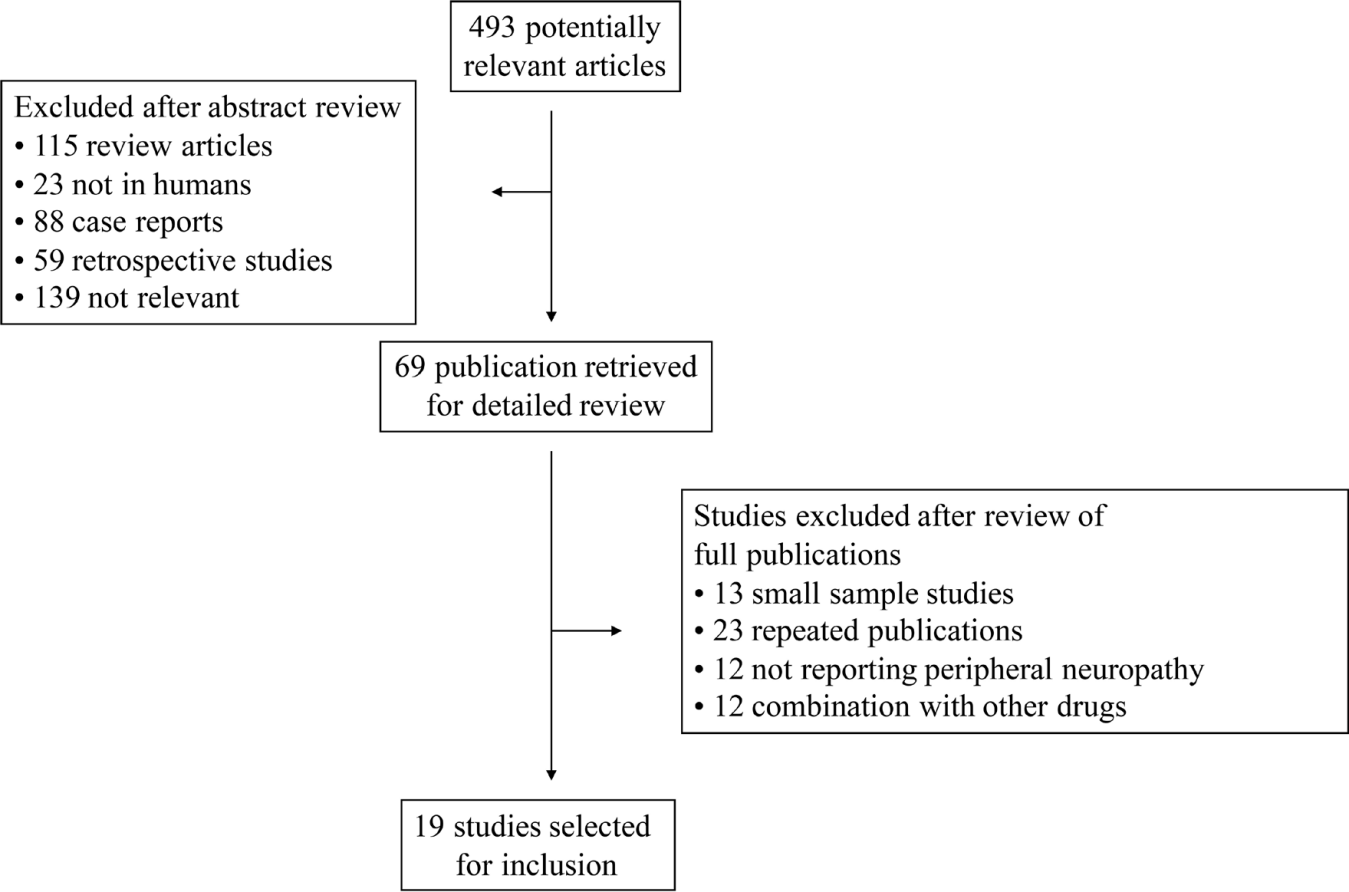
Selection process for the trials included in the meta-analysis

Our search yielded 361 potentially relevant studies on eribulin from PubMed and Embase. Among them, 345 were excluded after preliminary review (Figure 1). Eighteen studies were included for the final analysis. The search for ASCO abstracts yielded 132 studies, among which only 1 abstract met inclusion criteria. Altogether, we included 19 clinical trials in the final analysis (Table 1). The studies were published between 2009 and 2017. The baseline information of the 19 primary studies were shown in Table 1, including 3 phase III randomized controlled trials, 14 phase II trials, one phase IV trial, and 1 EAP (expanded access program). The sample size of the primary studies ranged from 51 to 951 patients (median sample size, 108 patients).

**Table 1:** Main characteristics and results of the eligible studies

Year	Study	Phase	Source	Disease	Trial	Drug	Calculation	Dose	All-grade	High-grade	Patients	Clinical Trial No.
2017	Watanabe [19]	4	Pubmed	Breast	Single-arm	Eribulin	Incidence	1.4 mg/m^2^ d1d8 Q3w	160	26	951	NCT01463891
2017	Park [20]	4	Pubmed	Breast	Single-arm	Eribulin	Incidence	1.4 mg/m^2^ d1d8 Q3w	27	2	101	NCT01961544
2016	Yardley [21]	2	Pubmed	Breast	Single-arm	Eribulin	Incidence	1.4 mg/m^2^ d1d8 Q3w	16	3	65	NCT01427933
2017	Kawai [22]	2	Pubmed	Sarcoma	Single-arm	Eribulin	Incidence	1.4 mg/m^2^ d1d8 Q3w	16	0	51	NCT01458249
2016	Schöffski [3]	3	Pubmed	Sarcoma	RCT	Eribulin	Incidence & RR	1.4 mg/m^2^ d1d8 Q3w	46	4	226	NCT01327885
						Dacarbazine			8	0	224	-
2016	Inoue [8]	2	Pubmed	Breast	Single-arm	Eribulin	Incidence	1.4 mg/m^2^ d1d8 Q3w	6	1	51	000006965
2016	Aftimos [23]	EAP	Pubmed	Breast	Single-arm	Eribulin	Incidence	1.4 mg/m^2^ d1d8 Q3w	65	10	154	NCT01240421
2015	Quinn [24]	2	ASCO	Urothelial	Single-arm	Eribulin	Incidence	1.4 mg/m^2^ d1d8 Q3w	67	NR	150	NCT00365157
2015	Kaufman [4]	3	Pubmed	Breast	RCT	Eribulin	Incidence & RR	1.4 mg/m^2^ d1d8 Q3w	149	38	544	NCT00337103
						Capecitabine			75	5	546	-
2014	McIntyre [9]	2	Pubmed	Breast	Single-arm	Eribulin	Incidence	1.4 mg/m^2^ d1d8 Q3w	32	11	56	NCT01268150
2013	Vahdat [5]	2	Pubmed	Breast	RCT	Eribulin	Incidence & RR	1.4 mg/m^2^ d1d8 Q3w	16	5	51	NCT00879086
						Ixabepilone			22	10	50	-
2012	Spira [25]	2	Pubmed	Lung	Single-arm	Eribulin	Incidence	1.4 mg/m^2^ d1d8 Q3w/Q4w	21	2	103	NA
2012	Gitlitz [26]	2	Pubmed	Lung	Single-arm	Eribulin	Incidence	1.4 mg/m^2^ d1d8 Q3w	20	2	66	NCT00400829
2012	de Bono [27]	2	Pubmed	Prostate	Single-arm	Eribulin	Incidence	1.4 mg/m^2^ d1d8 Q3w	15	3	108	NCT00278993
2012	Aogi [28]	2	Pubmed	Breast	Single-arm	Eribulin	Incidence	1.4 mg/m^2^ d1d8 Q3w	19	3	81	NCT00633100
2011	Schöffski [29]	2	Pubmed	Sarcoma	Single-arm	Eribulin	Incidence	1.4 mg/m^2^ d1d8 Q3w	43	4	127	NCT00413192
2011	Cortes [6]	3	Pubmed	Breast	RCT	Eribulin	Incidence & RR	1.4 mg/m^2^ d1d8 Q3w	174	41	503	NCT00388726
						TPC ^*^			45	5	247	-
2010	Cortes [30]	2	Pubmed	Breast	Single-arm	Eribulin	Incidence	1.4 mg/m^2^ d1d8 Q3w	95	17	291	NA
2009	Vahdat [31]	2	Pubmed	Breast	Single-arm	Eribulin	Incidence	1.4 mg/m^2^ d1d8 Q3w/Q4w	32	5	103	NA

Summary table of studies included in the meta-analysis. Abbreviations: N, number of patients; CI, confidence interval; RCT, randomized controlled trial; NR, not reported.

All studies reported the incidence of peripheral neuropathy associated with eribulin. The underlying malignancies include breast cancer, lung cancer, prostate cancer, urothelial cancer and sarcoma. For calculation of the RRs, 4 clinical trials were pooled [3–6]. The meta-analysis adheres to the guidelines of the Preferred Reporting Items for Systematic review and Meta-Analyses (PRISMA) statement [7].

### Incidence of peripheral neuropathy

**Figure 2:**
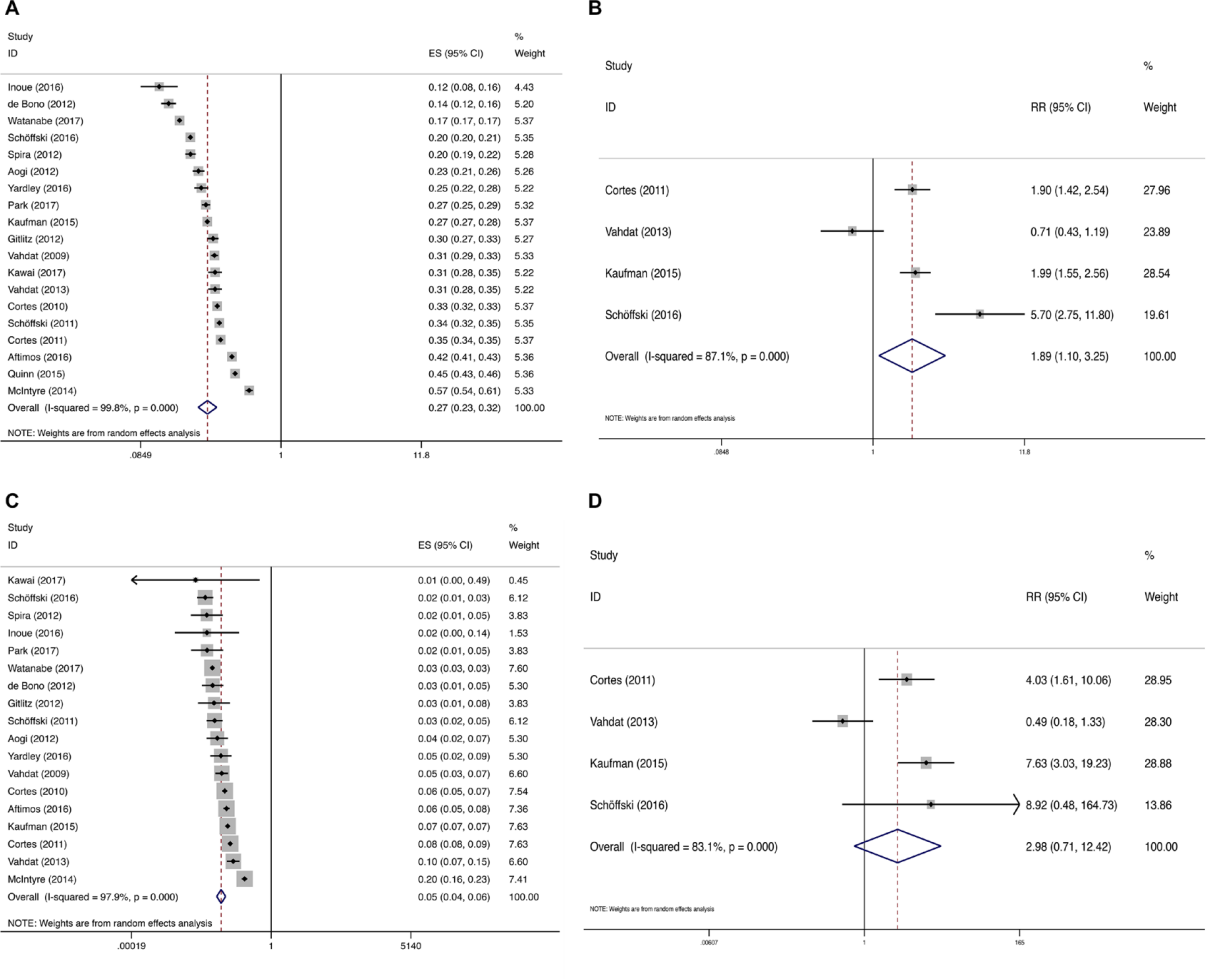
Forest plot for meta-analysis of relative risk and incidence of all-grade and high-grade peripheral neuropathy in cancer patients treated with eribulin Each study was shown by the name of the lead author and year of publication. The summary incidence and RR were also shown in the figure. Relative risk of eribulin-associated all-grade and high-grade peripheral neuropathy versus control from controlled trials of patients with cancer. Plots are arranged as follows: (**A**) Incidence of all-grade peripheral neuropathy; (**B**) Incidence of high-grade peripheral neuropathy; (**C**) Relative risk of eribulin-associated all-grade peripheral neuropathy vs control; (**D**) Relative risk of eribulin-associated high-grade peripheral neuropathy vs control.

The incidences of peripheral neuropathy of this meta-analysis were shown in Figure 2. Data for all-grade peripheral neuropathy included a total of 3,782 patients from 19 trials. Incidence of all-grade peripheral neuropathy ranged from 11.8% to 57.1% with the lowest incidence in a phase 2 trial by Inoue *et al.* [8], and the highest incidence in breast cancer patients [9]. The summary incidence of all-grade peripheral neuropathy was 27.5% (95% CI: 23.3% – 32.4%), according to the random-effects model (Heterogeneity test: *I*^2^ = 99.8%, *P* < 0.0001) (Figure 2A).

Eighteen trials reported the incidence of high-grade peripheral neuropathy, with incidence ranging from 0 to 19.64%. The highest incidence was reported in a phase 2 trial in breast cancer patients [9], and the lowest incidence in sarcoma patients [10]. The summary incidence of high-grade peripheral neuropathy was 4.7% (95% CI: 3.6%–6.2%), using a random effects model (Heterogeneity test: *I*^2^ = 97.9%, *P* < 0.0001) (Figure 2B).

**Figure 3:**
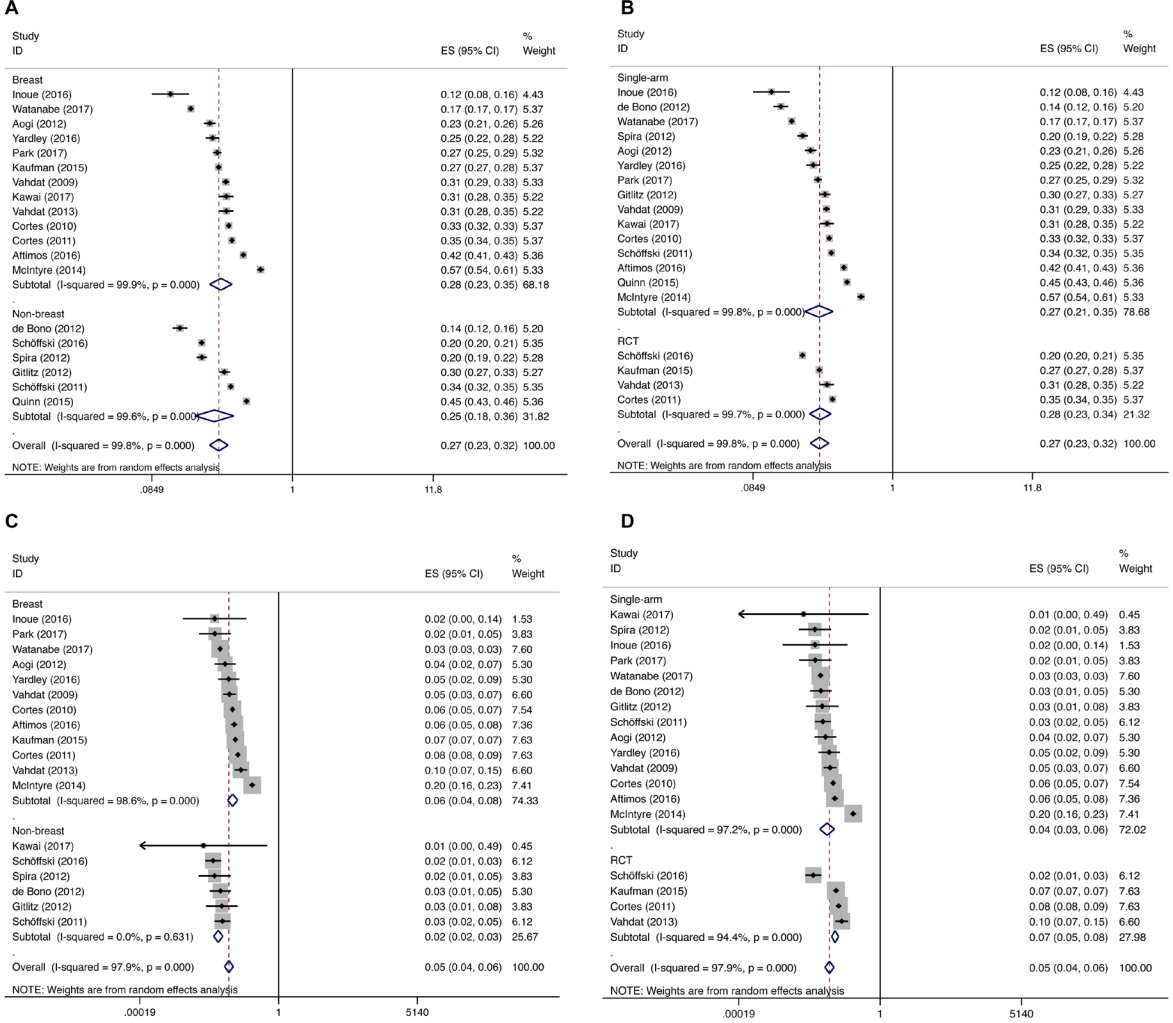
Subgroup analysis for incidence of all-grade and high-grade peripheral neuropathy Each study was shown by the name of the lead author and year of publication. The summary incidences were also shown in the figure. Plots are arranged as follows: (**A**) Incidence of all-grade peripheral neuropathy in breast vs non-breast cancer patients; (**B**) Incidence of all-grade peripheral neuropathy in breast vs non-breast cancer patients; (**C**) Incidence of all-grade peripheral neuropathy in single-arm vs RCT; (**D**) Incidence of high-grade peripheral neuropathy in single-arm vs RCT.

The trials were subgrouped by the underlying malignancy (breast vs non-breast cancer patients), and the incidence was calculated. All-grade incidence for breast and non-breast cancer patients was 28.5% (95% CI: 23.4%–34.7%) and 25.4% (95% CI: 18.1%– 35.7%), respectively. Breast cancer patients have a higher incidence of high-grade peripheral neuropathy than non-breast cancer patients (5.8%, 95% CI: 4.3%–8.0% and 2.4%, 95% CI: 1.8%–3.2%, breast vs non-breast cancer patients, respectively). The incidence of all-grade and high-grade peripheral neuropathy in non-breast cancer patients (lung cancer, prostate cancer, urothelial cancer and sarcoma) ranged from 13.89% to 44.67%, and 0 to 3.15%, respectively. However, due to the limited primary studies of each type of cancer involved, subgroup analysis of separate cancer type was not performed. The cancer type can partly explain the heterogeneity between the trials in terms of high-grade incidence, and the subgroup difference reached the level of statistical significance. We also calculated the differences in incidence according to study type (randomized controlled trial vs single-arm), and no significant differences were found (Figure 3). We conducted a random-effects meta-regression to quantify the effect of those factors, and the results indicated that the incidence of high-grade peripheral neuropathy varied with cancer type (breast vs non-breast cancer patients, *P* = 0.017), while all-grade incidence did not seem to be affected by cancer type (*P* = 0.568). Study type (randomized controlled trial vs single-arm study) and clinical trial phase did not affect all-grade and high-grade incidence (all *P* > 0.05).

### Relative risk of peripheral neuropathy

RR of peripheral neuropathy associated with eribulin compared with control was determined. The control arms included dacarbazine [3], capecitabine [4], ixabepilone [5], and TPC (treatment of physician’s choice) [6]. The pooled RR showed that eribulin increased the risk of all-grade peripheral neuropathy in cancer patients with a RR of 1.89, 95% CI: 1.10–3.25, suggesting a nearly two-fold risk for developing peripheral neuropathy with eribulin compared with control (Figure 2C). Significant heterogeneity was found (*I*^*2*^ = 87.1%; *P* < 0.0001). The RR for high-grade peripheral neuropathy was not increased (RR = 2.98, 95% CI: 0.71–12.42, Figure 2D) (*I*^*2*^ = 83.1%, *P* < 0.0001).

### Publication bias

**Figure 4:**
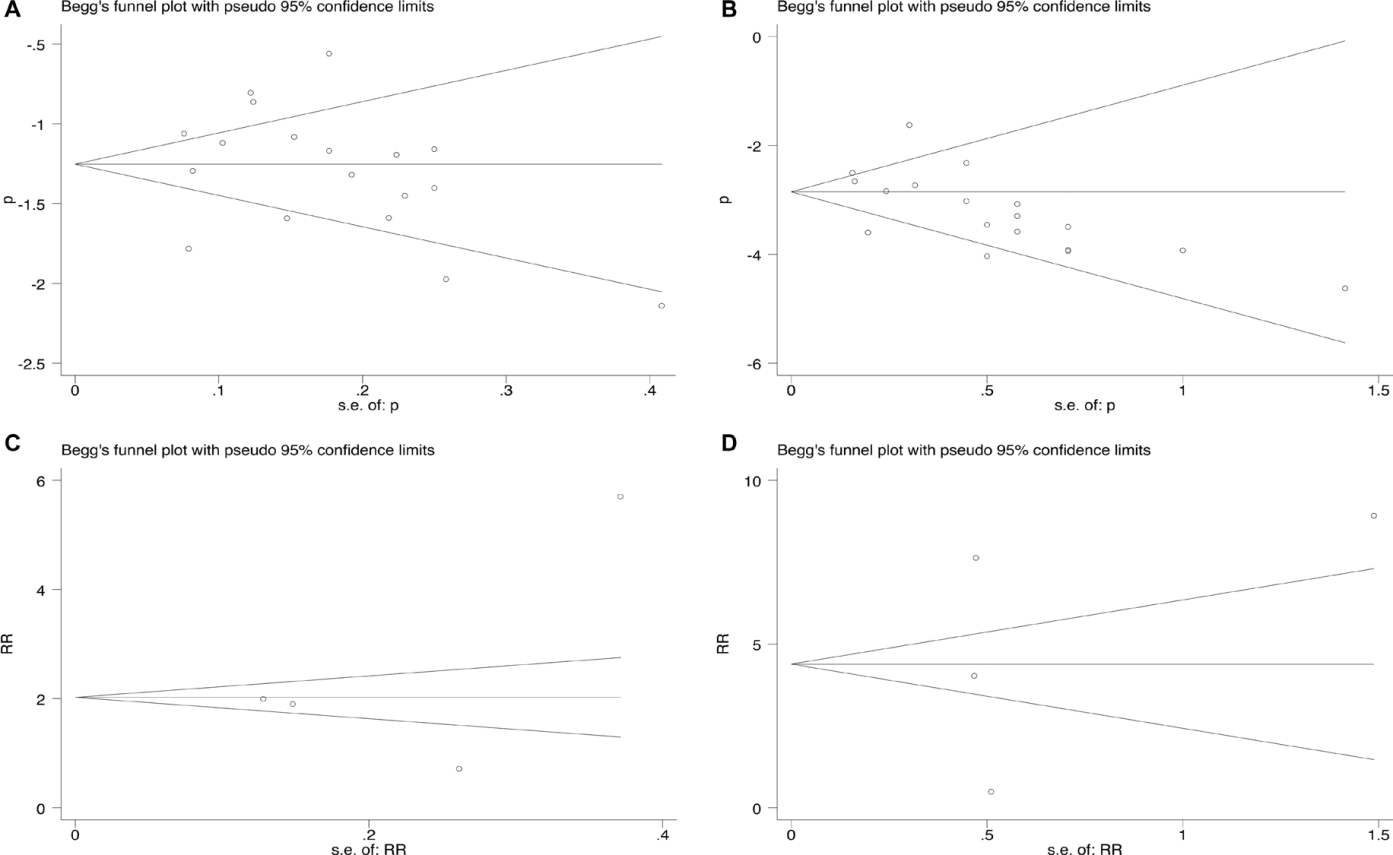
Funnel plot for studies included in the meta-analysis Each study was shown by the name of the lead author and year of publication. The summary incidences were also shown in the figure. Plots are arranged as follows: (**A**) Publication bias of studies of incidence of all-grade peripheral neuropathy; (**B**) Publication bias of studies of incidence of high-grade peripheral neuropathy; (**C**) Publication bias of studies of RR of all-grade peripheral neuropathy; (**D**) Publication bias of studies of high-grade peripheral neuropathy.

Eighteen studies reporting all-grade and high-grade peripheral neuropathy induced by eribulin resulted in an Egger’s test score of *P* = 0.894 and *P* = 0.072, respectively (Figure 4). Results for publication bias from trials investigating RR were also shown in Figure 4 (*P* = 0.534 and 0.789 for RR of all-grade and high-grade peripheral neuropathy, respectively).

### Sensitivity analysis

**Figure 5:**
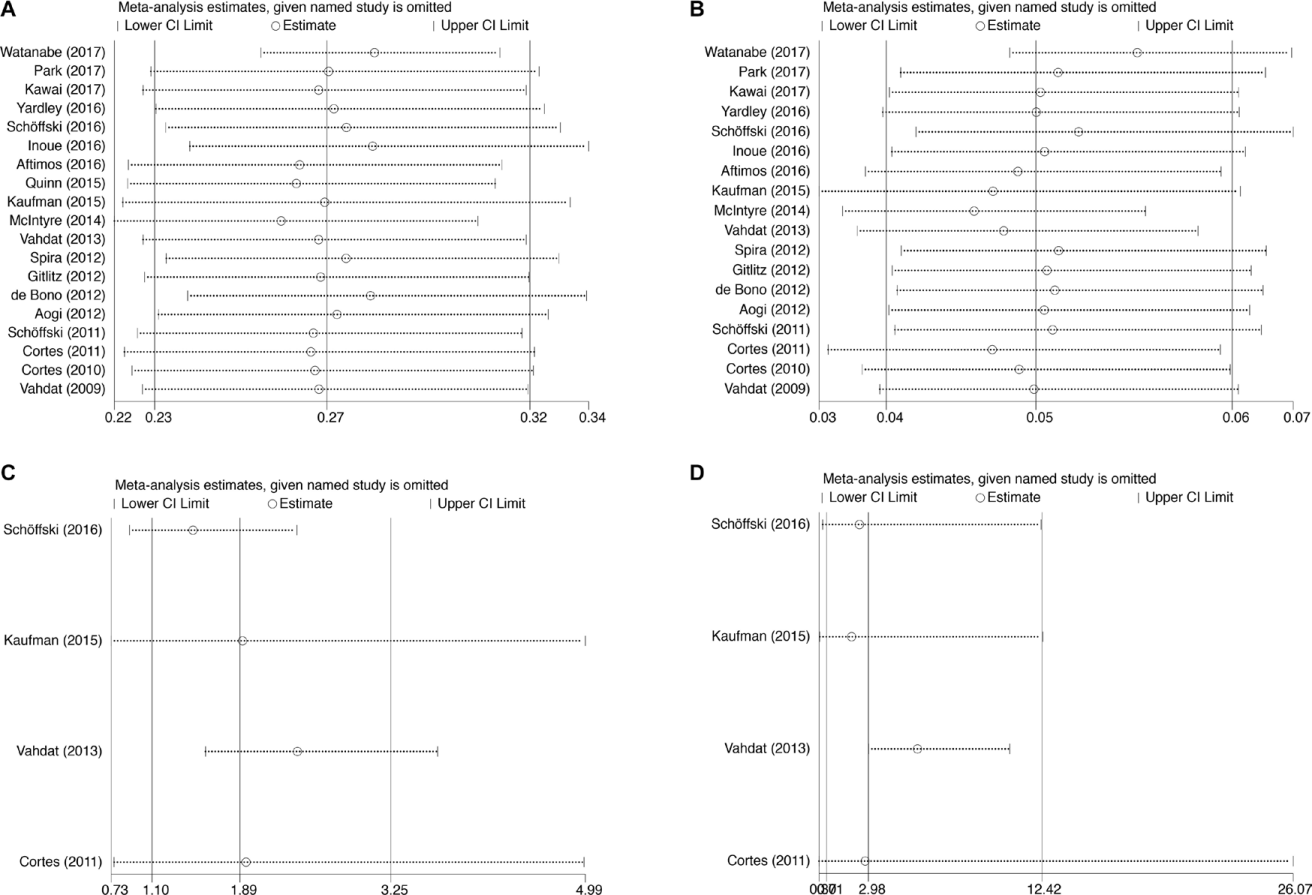
Sensitivity analysis for studies included in the meta-analysis Plots are arranged as follows: (**A**) Sensitivity analysis of incidence of all-grade peripheral neuropathy; (**B**) Sensitivity analysis of incidence of high-grade peripheral neuropathy; (**C**) Sensitivity analysis of RR of all-grade peripheral neuropathy; (**D**) Sensitivity analysis of RR of high-grade peripheral neuropathy.

Sensitivity analysis was performed to test the robustness and stability of our results. The significance estimate of pooled results was not influenced by omitting any single study (Figure 5).

## DISCUSSION

Eribulin mesylate is an halichondrin B analog, which binds to tubulin and microtubules. Eribulin was approved for the treatment of metastatic breast cancer patients, which is administered as a single agent at 1.4 mg/m^2^ IV on days 1 and 8 of a Q3W (every 3 weeks) cycle. The mechanism of eribulin is a novel action, which is distinct from other tubulin-targeted drugs, such as taxanes, epothilones, and vinca alkaloids [11]. Peripheral neuropathy is one of the non-hematological toxicities of eribulin. The underlying mechanism of eribulin-associated peripheral neuropathy is not entirely understood. The data on the summary incidence RR of eribulin-associated peripheral neuropathy are rather limited. We conducted a meta-analysis to determine the incidence and RR of peripheral neuropathy in patients receiving eribulin.

This is the first meta-analysis evaluating the incidence and RR of peripheral neuropathy associated with eribulin. In this meta-analysis, prospective clinical trials and expanded access programs of eribulin were included. The main finding of the present study is that RRs of peripheral neuropathy of eribulin compared to control were increased for all-grade (RR = 1.89, 95% CI: 1.10–3.25) but not for high-grade (RR = 2.98, 95% CI: 0.71–12.42). Risk of high-grade peripheral neuropathy was not increased, for which we suggest there might be several possible reasons. In primary studies, if the events are rare (ie, < 5%), they are often under-reported. Clinical trials are usually designed to investigate efficacy, but not specifically to address adverse events.

Our meta-analysis results demonstrated that eribulin is associated with an increased incidence of peripheral neuropathy. The overall incidence of all-grade and high-grade peripheral neuropathy was 28.6% (95% CI: 24.2–33.8%) and 4.7% (95% CI: 3.6–6.2%, respectively. We performed a subgroup analysis based on different tumor types. Considering the small number of events, the subgroup analysis can only be explained with caution. Indeed, the incidence of eribulin-associated peripheral neuropathy in breast cancer patients, the most common tumor type in our meta-analysis, seems to have a trend to increase in all-grade peripheral neuropathy (30.3% vs 25.4% for breast vs non-breast cancer patients, respectively), while statistically significant higher in high-grade peripheral neuropathy than non-breast cancers. The incidence and risk of peripheral neuropathy might be higher in the older and heavily treated population. Prior exposure to other neurotoxic agents complicates the risk for an individual patient. While breast cancer patients have a longer overall survival, patients receiving multiple lines of chemotherapy would have a higher chance of developing peripheral neuropathy. No significant difference was found between study design or trial phase of eribulin for all-grade and high-grade incidence of peripheral neuropathy. However, there is still the possibility of a real difference because of the limited number of trials and sample size of patient population included.

In addition to eribulin, other anti-neoplastic drugs, such as taxanes, bortezomib, cisplatin, also display dose-limiting toxicity of peripheral neuropathy [12, 13]. Depending upon the severity of the peripheral neuropathy, the offending agent is usually reduced, delayed, or discontinued. The manufacturer’s package insert also suggested to suspend the use of eribulin in patients who developed grade 3 or 4 peripheral neuropathy, until the resolution to grade 2 or less. Palliative care studies have been conducted to prevent or alleviate the symptom. Current management is mostly based on previous experiences and the severity of the symptom, which may be graded using the Common Terminology Criteria for Adverse Events (CTCAE) classification system. Few treatments are available for peripheral neuropathy. The ASCO guideline recommended treatment option for chemotherapy-induced peripheral neuropathy is duloxetine [14].

Our meta-analysis is not without limitations. One limitation of our meta-analysis is the lack of access to individual patient data, and these studies are conducted at various institutions with different baseline characteristics (Table 1). Secondly, a continuity correction of 0.5 subjects was applied, which might slightly overestimated the actual event rate. Thirdly, there were heterogeneity among primary clinical studies regarding tumor types and sample sizes.

Despite the above limitations, our meta-analysis is the first study to systematically quantify the incidence and RR of eribulin-associated peripheral neuropathy. The RRs of peripheral neuropathy of eribulin compared to control were increased for all-grade but not for high-grade. We observed a nearly two-fold RR of all-grade peripheral neuropathy with eribulin. High-grade peripheral neuropathy events were raised only slightly in the trend but not statistically, probably because of the limited sample size and events reported. Physicians should be aware of the possibility of increased peripheral neuropathy, especially in high-risk patients, and adverse events need careful monitoring, surveillance and reporting.

## MATERIALS AND METHODS

### Search strategy and study selection

An comprehensive search of PubMed database and Embase citations was conducted, with search terms “Eribulin”, “E7389”; AND “cancer”, “carcinoma”, “sarcoma”; AND “clinical trial”; AND “sensory neuropathy”, “peripheral neuropathy”, “chemotherapy induced peripheral neuropathy”, “CIPN”. The search was limited to published studies in English. Abstracts from ASCO were also searched. The upper date limit of June 2017 was applied, with no lower date limit. The clinical trial registration website (http://www.clinicaltrials.gov) was also searched. The manufacturer’s package insert of eribulin was also reviewed to obtain relevant information.

The inclusion criteria in this meta-analysis were: (1) prospective clinical trials conducted with cancer patients; (2) patients assigned to receive eribulin; (3) events available for peripheral neuropathy. Only the complete publication was included when multiple publications of the same trial were identified. Trials with relatively small number of patients (less than 50) were excluded. Phase I studies were excluded due to the different drug doses in these trials. Abstracts were read by two independent readers (LP and XY). Articles that could not be determined based on title and abstract were full-text reviewed.

### Study selection

Data extraction was performed by two authors (LP and XY) independently, and discrepancies were resolved by consensus. Peripheral neuropathy was reported in the toxicity profile of each study. Peripheral neuropathy were recorded according to versions 4.0 of the CTCAE of National Cancer Institute (http://ctep.cancer.gov/reporting/ctc_archive.html).

### Data analysis

Detailed information was collected from the primary studies, including the following items: first author, publication year, underlying malignancy, sample size, dose of eribulin, and control arm. If data were not reported, items were treated as “NR (not reported)”. The proportion of patients with peripheral neuropathy and 95% confidence interval (CI) were retrieved for each study. Studies which had a control arm were used to calculate RRs of peripheral neuropathy. For a study that reported zero event, we used the half-integer correction to calculate the RR and variance [15]. Authors of the primary studies were not contacted for additional data.

Heterogeneity was calculated employing the χ^2^-based *Q* test and *I*^*2*^ statistic [16]. Heterogeneity was considered statistically significant when *P*heterogeneity < 0.1 or *I*^2^ > 50%. Data were analyzed using a random effects model, if heterogeneity existed. Otherwise, a fixed effects model was used. An inverse variance statistical method was used to calculate the pooled incidence. We conducted a meta-regression analysis to test for variation in incidence estimates by other confounding factors. We performed subgroup analyses by different tumor type or trial design to explore the reasons for heterogeneity. *P* < 0.05 was considered significant. Publication bias was evaluated by using the Begg’s and Egger’s tests [17, 18]. Sensitivity analysis was performed by sequential omission of a single individual study to assess the stability and reliability of results. All calculations were performed by STATA version 14.0 (Stata Corporation, College Station, TX).
